# Stroboscopic balance training enhances dynamic stability and postural control in collegiate badminton players: a randomized controlled trial

**DOI:** 10.7717/peerj.21464

**Published:** 2026-06-26

**Authors:** Yixuan Wang, Hongzhang Lv, Zepeng Lu, Zhixiong Jiang, Guole Jiang, Yugui Wang

**Affiliations:** 1 Department of Physical Education and Military Training, Zhejiang Ocean University, Zhoushan, Zhejiang, China; 2 Wuyi University, Jiangmen, Guangdong, China; 3 Sports Coaching College, Beijing Sport University, Beijing, China; 4 College of Basic Military and Political Education, National University of Defense Technology, Changsha, Hunan, China

**Keywords:** Stroboscopic visual training, Dynamic postural stability, Balance control, Badminton athletes, Sensorimotor integration

## Abstract

**Background:**

Balance and landing stability are critical for performance in high-intensity sports such as badminton; however, conventional balance training may not adequately replicate the intermittent visual disturbances encountered during competition. Stroboscopic visual training (SVT), which intermittently occludes visual input to challenge sensory processing, has shown potential benefits but remains underexplored in badminton-specific settings. This study aimed to investigate the effects of SVT on static balance, dynamic balance, and landing stability in trained collegiate badminton athletes.

**Methods:**

A six-week randomized controlled trial was conducted in 20 male collegiate badminton players, who were randomly assigned to either an SVT group (*n* = 10) or a conventional balance training group (CON, *n* = 10). Both groups completed identical 30-min balance training sessions three times per week. The SVT group trained with stroboscopic eyewear in active flicker mode, whereas the CON group wore identical eyewear without visual occlusion. Pre- and post-intervention assessments included static balance (eyes-closed single-leg stance), dynamic balance (Y-Balance Test), and landing stability, using the Dynamic Postural Stability Index (DPSI) and center-of-pressure (COP) variables.

**Results:**

The SVT group demonstrated significantly greater improvements than the CON group in static balance, dynamic balance, and landing stability (*P* < 0.05). Specifically, the SVT group exhibited increased single-leg stance time, greater reach distances in the Y-Balance Test, and lower DPSI values, indicating improved postural stabilization after landing.

**Discussion:**

These findings suggest that incorporating SVT into training may be an effective strategy for enhancing postural control and landing stability in badminton athletes. From a practical perspective, these improvements may contribute to greater movement efficiency, enhanced landing control, and faster transitions during high-intensity play. Future studies should further investigate the underlying mechanisms and the transfer of these adaptations to sport-specific performance.

## Introduction

Badminton is a high-intensity racket sport characterized by technical and tactical demands (Phomsoupha & Laffaye, 2015; Tao, 2002), with a strong reliance on athletes’ balance and landing stability (He et al., 2025; Pasma et al., 2015). During competition, players are required to repeatedly perform highly dynamic and complex movements, such as lunge retrievals, rotational attacks, and jump smashes (Hong et al., 2014). Previous studies have demonstrated that enhanced balance improves the stability and precision of technical execution and contributes to greater footwork speed (Rouissi et al., 2018). Furthermore, superior landing stability enables more efficient transitions between offensive and defensive actions and facilitates rapid changes in direction. Therefore, the targeted development of balance and landing stability represents a fundamental element in badminton-specific training programs (Fong et al., 2007; McKeon & Hertel, 2008; Zhang et al., 2024).

Conventional balance training methods have been shown to improve athletes’ balance and landing stability; however, they are limited in replicating the fragmented visual information and complex dynamic environments encountered in actual competition (Guo et al., 2021; Malwanage, Senadheera & Dassanayake, 2022; McGuine & Keene, 2006). Traditional balance training typically includes static and dynamic exercises performed under full visual conditions, such as single-leg stance and unstable-surface training, without systematic manipulation of sensory input. In real match settings, athletes frequently experience intermittent visual disruptions due to the high speed of the shuttlecock, unpredictable opponent actions, and continuous head and body movements. Such conditions challenge the integration of sensory information and may compromise postural control during rapid movements. Vision plays a critical role in balance regulation by providing essential spatial and directional cues. When visual input is reduced or perturbed, the body relies more heavily on vestibular and proprioceptive systems, which may lead to decreased postural stability (Kim, Kim & Grooms, 2017; Kim et al., 2020; Miao et al., 2024; Pasma et al., 2015).

Stroboscopic visual training (SVT) is a novel training modality grounded in neuroscientific principles. It employs specialized eyewear to intermittently occlude visual input, thereby enhancing visual processing efficiency and strengthening perception–action coupling (Hoppeler, 2016). Previous studies have reported that SVT can improve reaction time, sensorimotor integration, and balance performance in various populations, including soccer players and individuals with ankle instability (Kim, Kim & Grooms, 2017; Lee et al., 2022; Zwierko et al., 2024). Despite these promising findings, evidence remains inconsistent regarding its effects on dynamic balance and landing stability, particularly in sport-specific contexts. Moreover, most existing studies have focused on general or non-specific athletic populations, with limited investigation in badminton, a sport characterized by rapid directional changes and frequent jump-landings that place unique demands on postural control.

Therefore, whether SVT can effectively enhance balance and landing stability in badminton athletes remains unclear. The Dynamic Postural Stability Index (DPSI) is widely used to assess an individual’s ability to stabilize the body following landing tasks, while center of pressure (COP) displacement provides additional insight into postural control by quantifying fluctuations in ground reaction forces during stabilization. Accordingly, the present study aimed to integrate SVT into a badminton-specific balance training program and to evaluate its effects on static balance, dynamic balance, and landing stability using DPSI and COP measures. The findings are expected to provide both theoretical insights and practical guidance for optimizing balance training in collegiate badminton athletes.

## Methods

### Participants

A two-way mixed-design analysis of variance (ANOVA) was planned, with group (SVT *vs.* conventional balance training group (CON) as the between-subjects factor and time (pre-test *vs.* post-test) as the within-subjects factor. An *a priori* sample size estimation was performed using G*Power software (version 3.1.9.7), with the group × time interaction specified as the primary effect of interest. The parameters were set as follows: medium effect size (*f* = 0.25), significance level (*α* = 0.05), and statistical power (1-*β* = 0.80). The analysis indicated that a minimum total sample of 20 participants was required, with equal allocation to each group (*n* = 10). This calculation was based on the primary outcome variables of the study (DPSI and COP). No formal adjustment for multiple outcome measures was applied in the sample size calculation, which should be considered when interpreting the results.

**Figure 1:**
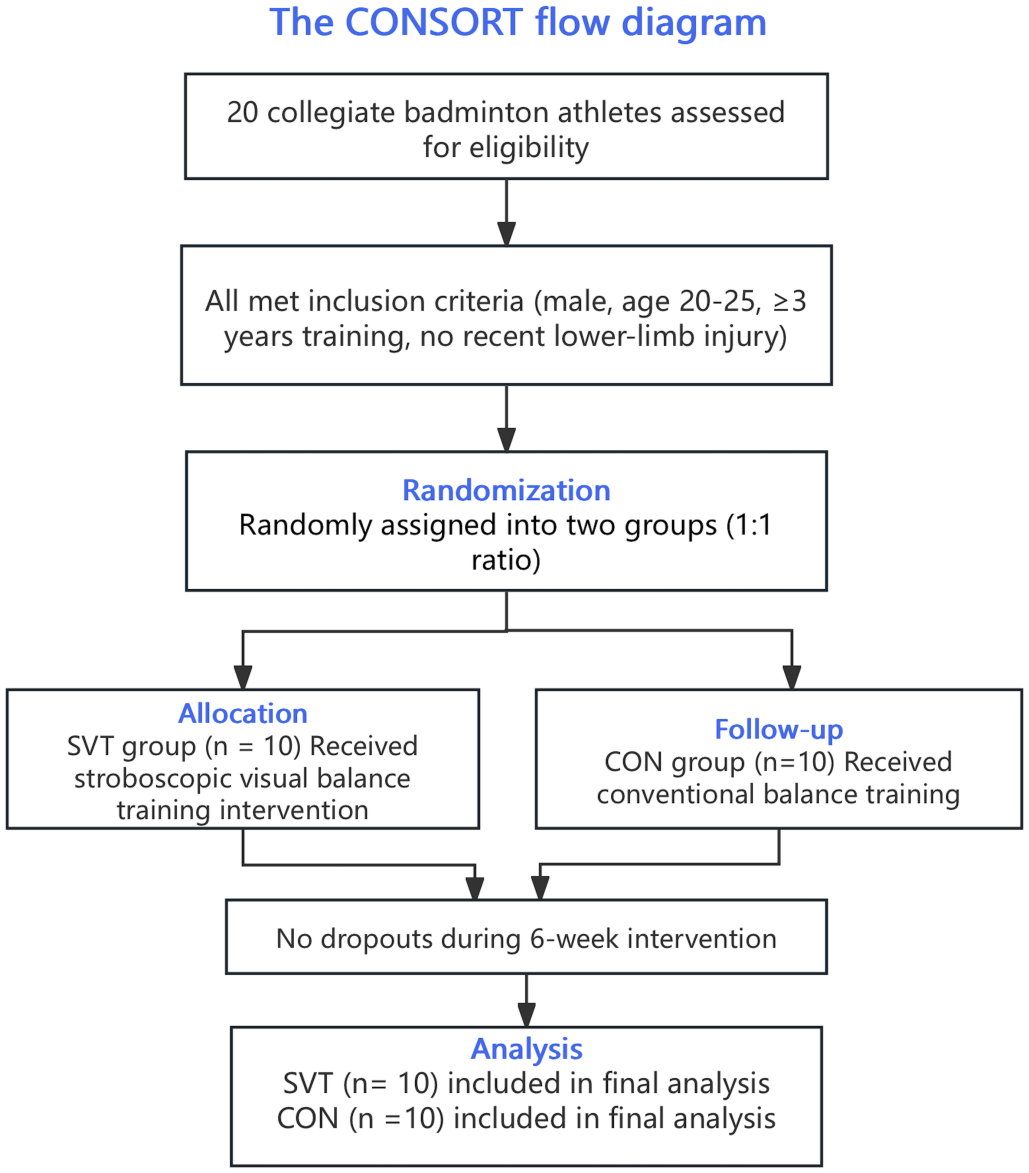
CONSORT flow diagram illustrating participant recruitment, allocation, follow-up, and analysis.

A total of 20 eligible collegiate badminton athletes were recruited, and all completed the intervention and testing. Figure 1 presents the Consolidated Standards of Reporting Trials (CONSORT) flow diagram of participant recruitment, allocation, follow-up, and analysis. In this diagram, allocation refers to group assignment, whereas follow-up refers to intervention monitoring throughout the 6-week training period.

Inclusion criteria were as follows: male athletes aged 20–25 years; a minimum of three years of specialized badminton training; participants were considered trained, competitive-level badminton athletes based on their training background and competitive experience; no lower-limb injuries within the previous three months; and no acute or chronic conditions affecting balance or performance.

Participants were screened for visual or ocular conditions that could contraindicate the use of stroboscopic eyewear through self-report and a pre-participation health screening conducted by the research team. Participants had not engaged in intensive competition preparation in the past three months and maintained consistent training routines. They were instructed to refrain from consuming substances (*e.g.*, alcohol or caffeine) that could influence neuromuscular performance. No additional participants were recruited or excluded during the study.

Although the sample size was relatively small, all participants were trained badminton athletes with a relatively high and homogeneous performance level. In sports science research involving trained or elite populations, smaller sample sizes are common due to strict inclusion criteria and limited availability. Moreover, the relatively homogeneous training background reduces inter-individual variability, which may increase the sensitivity for detecting training-related effects.

All participants voluntarily enrolled and provided written informed consent. This study was approved by the Research Ethics Committee of Beijing Sport University (Approval No. 2024486H), and all procedures were conducted in accordance with the Declaration of Helsinki.

### Experimental design and grouping

This study was designed as a randomized controlled trial. Following recruitment and screening, 20 male collegiate badminton athletes were enrolled. Participants were randomly assigned to groups using a computer-generated randomization sequence (1:1 allocation). Allocation was performed by an independent researcher not involved in testing or data analysis. Participant demographic characteristics are presented in Table 1.

**Table 1:** Demographic characteristics of participants (Mean ± SD).

**Variable**	**SVT group (*n* = 10)**	**CON group (*n* = 10)**	***P*-value**
Age (years)	22.50 ± 0.85	22.40 ± 0.89	>0.05
Height (cm)	178.20 ± 3.12	177.80 ± 2.94	>0.05
Weight (kg)	74.60 ± 5.23	73.80 ± 4.87	>0.05
Training years	5.10 ± 1.23	5.30 ± 1.15	>0.05

**Notes.**

No significant differences were found between groups at baseline (all *P* > 0.05).

Both groups engaged in balance training three times per week, with each session lasting 30 min. The SVT group performed the balance training protocol while wearing stroboscopic eyewear with the flicker mode activated. The CON group underwent the same training while wearing the same eyewear in the powered-off state (*i.e.,* lenses remained fully transparent) throughout each session. This approach controlled for potential confounding effects related to wearing the device itself (*e.g.*, weight, minor visual obstruction) and ensured a single-blind design. This study employed a single-blind design. Participants were not informed of the specific purpose of the stroboscopic intervention, and both groups wore identical eyewear to minimize expectation bias. Outcome assessors and data analysts were blinded to group allocation throughout the testing and analysis phases.

No significant differences were observed between groups in baseline characteristics such as age, height, body mass, training experience, and competition level (*P* >0.05); detailed information is presented in Table 1. No participants dropped out during the study, and all completed the entire training intervention and testing procedures.

### Intervention protocol and testing procedures

Both groups completed balance training sessions simultaneously on Mondays, Wednesdays, and Fridays, with each session lasting 30 min. The training protocol was adapted from established balance training programs emphasizing dynamic postural control and sensorimotor integration (McKeon & Hertel, 2008). The program combined unstable-surface exercises and jump-stabilization tasks. Training difficulty was progressively increased over the six-week intervention by modifying task complexity (*e.g.*, static to dynamic tasks), reducing base of support stability (*e.g.*, bilateral to single-leg stance), and increasing movement speed and coordination demands. Participants were required to successfully complete each level before progressing to the next, ensuring a structured progression consistent with previous balance training protocols. Participants in both the SVT and CON groups wore stroboscopic eyewear during training. Additionally, all athletes participated in a standardized technical–tactical training program five times per week, with each session lasting approximately two hours and focusing on footwork, stroke techniques, and match simulations. The stroboscopic eyewear (EYESTROBE, Senaptec Inc., Beaverton, OR, USA, in collaboration with Zhejiang Shanmu Technology Co., China) was used during training. The eyewear consists of liquid crystal lenses that alternately switch between transparent and opaque states to intermittently disrupt visual input. The flicker frequency was set at three Hz, with a transparent phase of 0.10 s and an opaque phase of 0.23 s per cycle. Participants in the SVT group were instructed to wear and activate the eyewear throughout each training task and were allowed to remove it during rest intervals between exercises. Testing was conducted at baseline and within three days following the completion of the six-week training program (see Table 2 for details of the training protocols for the SVT and CON groups). To minimize fatigue-related effects, participants were instructed to avoid high-intensity exercise for at least 24 h prior to testing. All assessments were performed in a laboratory setting, with ambient temperature maintained at 20–25 °C and relative humidity at 40–50%. Researchers provided standardized verbal instructions and allowed participants one to two familiarization trials. Athletes completed a standardized warm-up and were encouraged to exert maximal effort during testing. Rest intervals of 5–10 min were provided between tests, and each test was administered within a consistent time window. Training adherence was strictly monitored by the research team. Attendance was recorded for each session, and all training sessions were supervised by qualified instructors. Participants were required to complete all prescribed sessions to be included in the final analysis. No dropouts or missed sessions were recorded during the intervention period. Training intensity was standardized across participants, and all sessions followed identical protocols to minimize inter-individual variation.

**Table 2:** Training protocols for SVT and CON groups.

Training content	Weeks 1–2	Weeks 3–4	Weeks 5–6
Single-leg balance	Hard-ground stance, arms crossed (1 min × 3 sets)	Foam pad stance, arms crossed (1 min × 3 sets)	Foam pad stance (30 s) + 20 medicine-ball catches (2 kg, 3 sets)
BOSU single-leg jumps	Continuous vertical hops (12 reps × 3 sets) + Lateral hops (12 reps × 3 sets)	Continuous hops with dumbbells (5 kg, 12 reps × 3 sets) + Lateral hops with dumbbells (5 kg, 12 reps × 3 sets)	Cross-shaped hops with dumbbells (5 kg, 12 reps × 3 sets)
Stabilization reach jumps	45 cm multi-directional reach (12 reps × 3 sets)	60 cm multi-directional reach (12 reps × 3 sets)	45 cm + hurdle multi-directional reach (12 reps × 3 sets)
Unpredictable single-leg hops	One hop every 3 s (9 reps × 3 sets)	One hop every 1 s (9 reps × 3 sets)	One hop every 1 s onto random 10 cm box (9 reps × 3 sets)

Training load was controlled by ensuring that all participants performed the same exercises, with identical duration, frequency, and progression criteria under direct supervision. Progression to more challenging tasks was permitted only after successful completion of the preceding level, thereby maintaining consistency in training stimulus across participants. Although no quantitative load metrics (*e.g.*, rating of perceived exertion) were recorded, adherence to the standardized protocol and continuous supervision ensured comparable training exposure between groups.

### Measurements

The dominant leg was defined based on the participant’s preferred leg during badminton play. For consistency in reporting, balance performance tests (*e.g.*, Y-Balance Test) were analyzed using dominant and non-dominant legs, whereas landing stability tasks (DPSI and COP) were reported according to the supporting leg (left *vs.* right).

#### Single-leg eagle stance test

**Figure 2:**
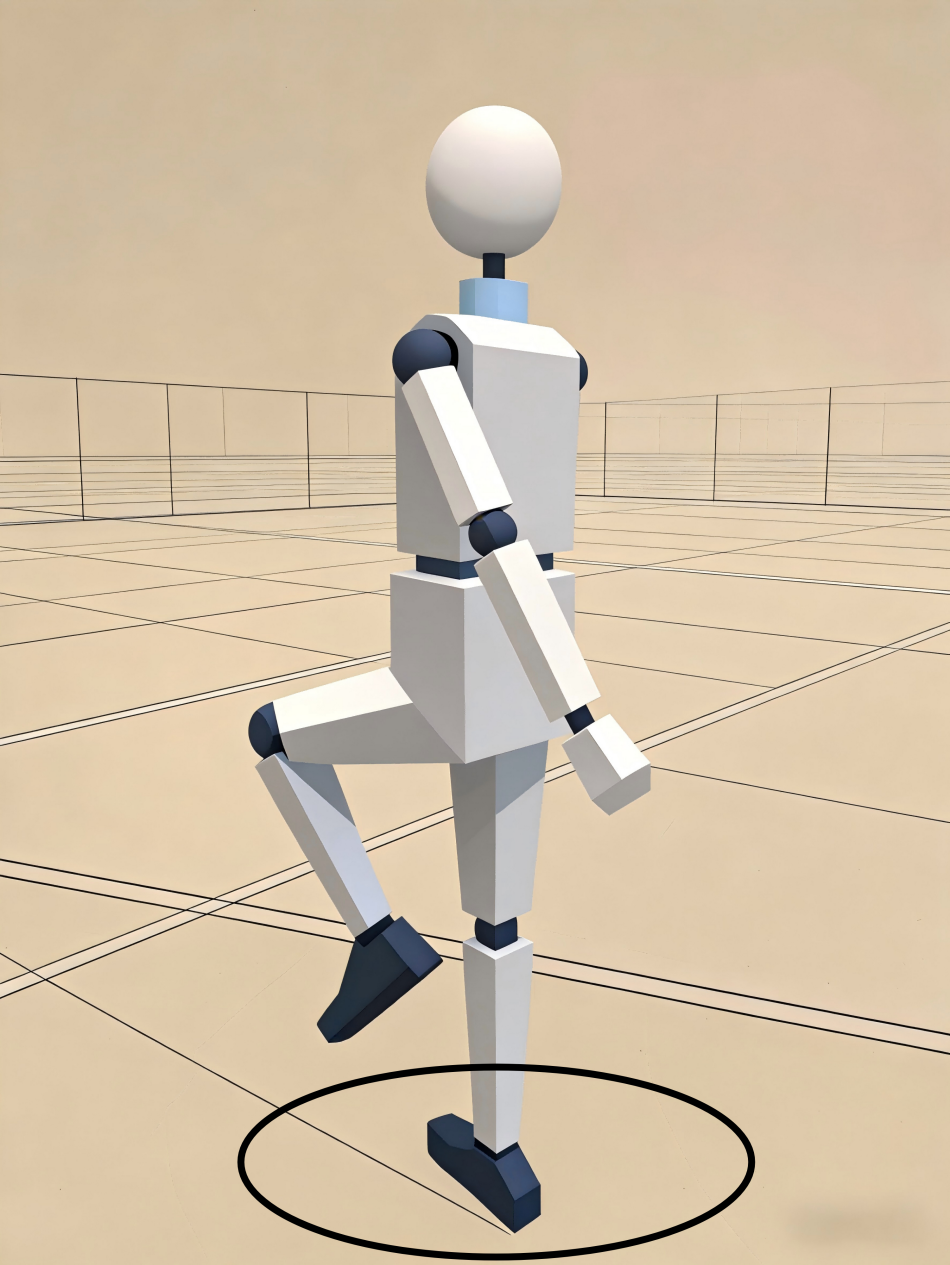
Schematic diagram of the blindfolded stepping-in-place test.

Static balance was assessed using a single-leg stance test under eyes-closed conditions (Zhang et al., 2023). Participants stood on one leg with their arms crossed over the chest, while the non-supporting leg was flexed at the knee. The test was initiated by a standardized verbal command. Timing began once the participant closed both eyes and achieved a stable posture. Timing was terminated when the participant opened one or both eyes, moved the stance foot, or lost balance. Each leg was tested three times, and the mean value was used for analysis. The dominant leg was determined based on the participant’s preferred leg during badminton play. The testing posture and experimental setup for the single-leg eagle stance assessment are illustrated in Fig. 2.

#### Y-balance test

Dynamic balance was assessed using the Y-Balance Test (YBT), a reliable and valid tool for evaluating lower-limb balance performance (Gribble, Hertel & Plisky, 2012; Plisky et al., 2009). Prior to testing, lower limb length was measured from the anterior superior iliac spine to the distal tip of the medial malleolus. This measurement was used to normalize reach distances and minimize the influence of anthropometric differences between participants. Participants stood barefoot on the testing platform with the dominant leg as the stance leg. Upon a standardized verbal signal, the contralateral leg was used to reach maximally in the anterior, posteromedial, and posterolateral directions. Each participant performed three trials in each direction. Trials were considered invalid if balance was lost, hands were removed from the hips, or the stance foot moved. The maximum reach distance in each direction was recorded and normalized to limb length. A composite score was calculated as:

\begin{eqnarray*}\text{Composite Score}= \frac{(\mathrm{ANT}+\mathrm{PM}+\mathrm{PL})}{(3\times \text{limb length})} \times 100. \end{eqnarray*}

#### Closed-eye on-the-spot stepping test

Postural orientation under visual deprivation was assessed using the eyes-closed stepping in place test (Zwierko et al., 2024). Participants stood within a marked circular area (diameter = 40 cm). Upon a standardized verbal signal, they were instructed to March in place with eyes closed at a controlled cadence. Step frequency (cadence) was defined as the number of steps performed per minute. Cadence was monitored by counting the number of steps performed within a fixed time interval and subsequently converted to steps per minute. Controlling cadence allowed for standardization of movement speed across participants and reduced variability in motor performance. The test was terminated when the participant stepped on or beyond the boundary or opened their eyes. Each participant performed three trials, and the best performance was retained for analysis.

#### Dynamic postural stability test and center of pressure COP indices

The Dynamic Postural Stability Index (DPSI) and center of pressure (COP) were assessed using a force platform (Kistler, Switzerland) with a sampling frequency of 1,000 Hz. Participants performed single-leg landing tasks following forward and lateral jumps. Each participant started from a standardized bilateral stance behind a marked take-off line and was instructed to jump forward or laterally and land on a single leg on the force platform. Upon landing, participants were required to stabilize as quickly as possible and maintain balance for a specified duration without additional hopping or foot adjustment. Trials were considered invalid if participants lost balance, touched the ground with the non-supporting foot, or failed to maintain the required posture. Each task was performed multiple times, and valid trials were averaged for analysis. The DPSI was calculated based on ground reaction force data following the method described by Wikstrom et al. (2005):

\begin{eqnarray*}DPSI= \frac{( \frac{\sqrt{\sum { \left( 0-GR{F}_{x} \right) }^{2}+\sum { \left( 0-GR{F}_{y} \right) }^{2}+\sum (0-GR{F}_{z})^{2}}}{number~of~data~points} )}{BW} \end{eqnarray*}

where GRFx, GRFy, and GRFz represent the ground reaction forces in the mediolateral, anterior–posterior, and vertical directions, respectively, BW represents body weight, and n represents the number of data points.

To enhance ecological validity, two additional jump-stabilization tasks were incorporated into the testing protocol: forward single-leg lunging jumps and lateral single-leg lunging jumps. Assessments were conducted before and after the six-week intervention period.

Participants stood behind a take-off line positioned at 40% of their body height. A hurdle was placed midway between the take-off line and the force plate, with a height of 30 cm for forward jumps and 15 cm for lateral jumps.

Upon a standardized verbal signal, participants performed lunging jumps over the hurdle and landed on the designated leg on the force plate. After landing, they were instructed to stabilize as quickly as possible, place their hands on their hips, and maintain a stable position for 10 s.

Each participant completed three valid trials for each condition, resulting in a total of 12 trials.

Center of pressure (COP) displacement in the anterior–posterior (AP) and mediolateral (ML) directions was calculated to evaluate postural control during the stabilization phase. COP-related variables were computed using standard biomechanical methods, consistent with previous studies (Sarvari et al., 2024).

Where COPx and COPy represent the coordinates of the center of pressure in the anterior–posterior and mediolateral directions, respectively, and n represents the number of data points.

A trial was considered invalid if any of the following conditions occurred:

(1) failure to stabilize after landing, accompanied by excessive sway;

(2) repeated hopping on the stance leg following landing;

(3) failure to clear the hurdle at the designated height;

(4) contact of the non-testing leg with the ground or surrounding area;

(5) removal of the hands from the hips for more than 5 s.

The reliability and validity of DPSI and COP measurements have been established in previous biomechanical studies (Wikstrom et al., 2005).

\begin{eqnarray*}CO{P}_{ML}& =\sum _{n=1}^{N-1}\sqrt{(CO{P}_{y} \left( n+1 \right) -CO{P}_{y}(n))^{2}} \end{eqnarray*}

\begin{eqnarray*}CO{P}_{AP}& =\sum _{n=1}^{N-1}\sqrt{(CO{P}_{x} \left( n+1 \right) -CO{P}_{x}(n))^{2}}. \end{eqnarray*}

### Statistical analysis

All statistical analyses were performed using SPSS version 25.0 (IBM Corp., Armonk, NY, USA). Continuous variables with normal distribution were expressed as mean ± standard deviation (Mean ± SD). Prior to inferential analysis, the assumptions of normality and homogeneity of variance were assessed using the Shapiro–Wilk test and Levene’s test, respectively. All variables met the assumptions for parametric analysis. Kinematic parameters were analyzed using a two-way mixed-ANOVA, with group (SVT *vs.* CON) as the between-subjects factor and time (pre- *vs.* post-training) as the within-subjects factor. When a significant interaction effect was observed, simple effects were further analyzed for each factor. In the absence of interaction, only the main effects were examined.

In addition to *P*-values, 95% confidence intervals (95% CI) and effect sizes were reported. Effect sizes were expressed as partial eta squared (*η*^2^) to indicate the magnitude of the observed effects, with thresholds of 0.01, 0.06, and 0.14 representing small, medium, and large effects, respectively. Cohen’s d was calculated to assess the magnitude of within- and between-group changes.

Effect size interpretation followed conventional thresholds: *d* < 0.2 (trivial), 0.2–0.6 (small), 0.6–1.2 (moderate), 1.2–2.0 (large), and *d* > 2.0 (very large). Exact *P*-values are reported where applicable, and statistical significance was set at *P* < 0.05 for all tests.

## Results

### Single-leg eagle stance with eyes closed

ANOVA results revealed a significant main effect of time on the left-leg test (*F* (1, 18) = 42.81, *P* <  0.001, *η*^2^ = 0.70). However, no significant group × time interaction was observed (*F* (1, 18) = 3.09, *P* = 0.096, *η*^2^ = 0.15). *Post hoc* comparisons indicated that the SVT group showed a significant improvement from pre- to post-intervention (mean difference = −1.630, *P* < 0.001, 95% CI [−2.087, −1.173], ES = 0.35), whereas no significant change was observed in the CON group (mean difference = −0.382, *P* = 0.096, 95% CI [−0.839, 0.075], ES = 0.17).

For the right-leg test, a significant main effect of time was observed (*F* (1, 18) = 26.20, *P* <  0.001, *η*^2^ = 0.59), whereas the group × time interaction was not significant. *Post hoc* comparisons indicated that both groups showed significant improvements following the intervention (SVT: mean difference = −1.340, *P* < 0.001, 95% CI [−1.954, −0.726], ES = 0.34; CON: mean difference = −0.774, *P* = 0.016, 95% CI [−1.388, −0.160], ES = 0.26).

### Y-balance test results

For the dominant leg, significant main effects of time (*F* (1, 18) = 46.86, *P* < 0.001, *η*^2^ = 0.72) and a significant group × time interaction (*F* (1, 18) = 6.74, *P* = 0.018, *η*^2^ = 0.27) were observed. *Post hoc* comparisons revealed that both groups showed significant improvements following the intervention (SVT: mean difference = −11.924, *P* < 0.001, 95% CI [−19.350, −10.498], ES = 2.02; CON: mean difference = −5.469, *P* = 0.018, 95% CI [−9.895, −1.043], ES = 1.44). Notably, the magnitude of improvement was greater in the SVT group compared with the CON group.

For the non-dominant leg, significant main effects of time (*F* (1, 18) = 52.46, *P* < 0.001, *η*^2^ = 0.75) and group (*F* (1, 18) = 6.68, *P* = 0.019, *η*^2^ = 0.27), as well as a significant group × time interaction (*F* (1, 18) = 9.47, *P* = 0.006, *η*^2^ = 0.35), were observed. *Post hoc* comparisons revealed that both groups showed significant improvements following the intervention (SVT: mean difference = −12.393, *P* < 0.001, 95% CI [−16.027, −8.759], ES = 1.93; CON: mean difference = −5.323, *P* = 0.006, 95% CI [−8.957, −1.689], ES = 0.95). Furthermore, between-group comparisons indicated that the SVT group demonstrated significantly greater improvements than the CON group (mean difference = 3.371, *P* = 0.019, 95% CI [0.631–6.111]).

### Closed-eye on-the-spot stepping test

A significant main effect of time was observed (*F* (1, 18) = 47.27, *P* < 0.001, *η*^2^ = 0.72), whereas neither the group effect (*F* (1, 18) = 0.05, *P* = 0.823, *η*^2^ = 0.003) nor the group × time interaction was significant. *Post hoc* comparisons revealed a significant improvement from pre- to post-intervention (mean difference = −0.980, *P* < 0.001, 95% CI [−1.279, −0.681]). Both groups demonstrated comparable magnitudes of improvement, with small effect sizes observed (SVT: ES = 0.37; CON: ES = 0.40). Descriptive statistics of balance performance indicators are presented in Table 3.

**Table 3:** Descriptive statistics of balance performance indicators (Mean ± SD).

**Variable**	**SVT pre**	**SVT post**	**CON Pre**	**CON Post**	**Time** (***P* value,***η*^2^)	**Group × Time** (***P* value,***η*^2^)
Single-leg eagle stance (non-dominant leg, *s*)	12.17 ± 4.71	13.80 ± 4.47^*^	11.90 ± 2.08	12.28 ± 2.29	*P* < 0.05, *η*^2^= 0.70	*P* < 0.05, *η*^2^= 0.48
Single-leg eagle stance (dominant leg, *s*)	14.74 ± 4.17	16.08 ± 3.71^*^	15.29 ± 2.69	16.06 ± 2.48	*P* < 0.05, *η*^2^= 0.59	*P* = 0.19, *η*^2^= 0.09
Y-Balance (dominant leg, %)	84.08 ± 6.02	96.00 ± 4.69^*^	85.75 ± 5.98	92.34 ± 3.37^*^	*P* < 0.05, *η*^2^= 0.72	*P* < 0.05, *η*^2^= 0.36
Y-Balance (non-dominant leg, %)	81.95 ± 2.57	90.34 ± 5.37^*^^#^	82.11 ± 3.37	86.67 ± 5.83^*^	*P* < 0.05, *η*^2^= 0.75	*P* < 0.05, *η*^2^= 0.32
Closed-eye stepping test (*s*)	12.01 ± 3.30	13.20 ± 3.10	11.95 ± 1.91	12.72 ± 1.92	*P* < 0.01, *η*^2^= 0.16	*P* = 0.16, *η*^2^= 0.11

**Notes.**

* Significant difference compared with pre-test (*P* <  0.05).

# Significant difference compared with CON group (*P* < 0.05).

### Dynamic postural stability test

In the DPSI test, significant main effects of time were observed across multiple jump directions (all *P* < 0.05). Detailed DPSI outcomes for each task are presented in Table 4.

**Table 4:** DPSI and COP outcomes across tasks (Mean ± SD).

	**Task/variable**	**SVT pre**	**SVT post**	**CON pre**	**CON post**	**Time** (***P -*value,***η*^2^)	**Group × Time (*P -*value,***η*^2^)
DPSI	Right-leg forward jump	1.14 ± 0.06	1.01 ± 0.09^*^^#^	1.13 ± 0.31	1.09 ± 0.04^*^	*P* < 0.05, *η*^2^= 0.40	*P* < 0.05, *η*^2^= 0.07
	Left-leg forward jump	1.20 ± 0.06	1.09 ± 0.10	1.19 ± 0.06	1.15 ± 0.05	*P* < 0.05, *η*^2^= 0.80	*P* = 0.08, *η*^2^= 0.09
	Right-leg lateral jump (to right)	1.26 ± 0.18	1.09 ± 0.12^*^	1.27 ± 0.03	1.23 ± 0.09	*P* < 0.05, *η*^2^= 0.19	*P* < 0.05, *η*^2^= 0.13
	Right-leg lateral jump (to left)	1.03 ± 0.04	0.94 ± 0.10^*^	1.03 ± 0.15	0.95 ± 0.13^*^	*P* < 0.05, *η*^2^= 0.41	*P* < 0.05, *η*^2^= 0.10
	Rotational jump	0.92 ± 0.09	0.82 ± 0.05	0.99 ± 0.12	0.87 ± 0.15^*^	*P* < 0.05, *η*^2^= 0.20	*P* = 0.44, *η*^2^= 0.03
COP (*mm*)	Right-leg forward jump (AP)	39.07 ± 8.42	36.20 ± 7.03^*^	39.40 ± 5.91	36.65 ± 5.32^*^	*P* < 0.05, *η*^2^= 0.19	*P* = 0.90, *η*^2^= 0.01
	Right-leg forward jump (ML)	49.34 ± 10.57	46.35 ± 9.94^*^	49.27 ± 9.45	46.99 ± 8.17^*^	*P* < 0.05, *η*^2^= 0.40	*P* < 0.05, *η*^2^= 0.07
	Right-leg lateral jump (AP)	42.37 ± 7.26	39.12 ± 7.19^*^	42.28 ± 9.00	40.45 ± 8.92^*^	*P* < 0.05, *η*^2^= 0.49	*P* < 0.05, *η*^2^= 0.08
	Right-leg lateral jump (ML)	42.93 ± 8.81	40.22 ± 8.03^*^	41.16 ± 11.12	41.16 ± 11.13^*^	*P* < 0.05, *η*^2^= 0.19	*P* = 0.53, *η*^2^= 0.02
	Left-leg lateral jump (AP)	36.29 ± 7.95	33.25 ± 7.67^*^	36.13 ± 8.20	33.63 ± 9.09^*^	*P* < 0.05, *η*^2^= 0.74	*P* = 0.50, *η*^2^= 0.03
	Left-leg lateral jump (ML)	45.87 ± 6.72	43.19 ± 6.31^*^	46.86 ± 9.62	44.28 ± 10.23^*^	*P* = 0.50, *η*^2^= 0.03	*P* < 0.05, *η*^2^= 0.18
	Rotational jump (AP)	44.69 ± 8.19	37.87 ± 6.66	43.44 ± 11.51	41.37 ± 11.62^*^	*P* < 0.05, *η*^2^= 0.18	*P* < 0.05, *η*^2^= 0.45
	Rotational jump (ML)	57.51 ± 8.52	52.63 ± 8.21	55.51 ± 11.78	53.60 ± 11.35^*^	*P* < 0.05, *η*^2^= 0.12	*P* < 0.05, *η*^2^= 0.31

**Notes.**

* Significant difference compared with pre-test (*P* < 0.05).

# Significant difference compared with CON group (*P* < 0.05).

AP anterior–posterior ML mediolateral

Several tasks also demonstrated significant group × time interactions, with a notable interaction observed in the right-leg forward jump (*F* (1, 18) = 13.70, *P* = 0.002, *η*^2^ = 0.43). *Post hoc* comparisons for this task revealed that both groups showed significant improvements following the intervention (SVT: mean difference = −0.149, *P* < 0.001, 95% CI [−0.183, −0.115], ES = 1.66; CON: mean difference = −0.044, *P* = 0.013, 95% CI [−0.078, −0.010], ES = 0.21). The magnitude of improvement was greater in the SVT group compared with the CON group.

For the left-leg forward jump, a significant main effect of time was observed (*F* (1, 18) = 16.69, *P* <  0.001, *η*^2^ = 0.48), whereas neither the group effect (*F* (1, 18) = 1.00, *P* = 0.332, *η*^2^ = 0.05) nor the group × time interaction was significant. *Post hoc* comparisons indicated a significant improvement from pre- to post-intervention (mean difference = 0.076, *P* < 0.001, 95% CI [0.037–0.115]). Both groups demonstrated comparable improvements over time (SVT: ES = 1.33; CON: ES = 0.72).

For the rotational jump, a significant main effect of time was observed (*F* (1, 18) = 50.50, *P* < 0.001, *η*^2^ = 0.74), whereas neither the group effect (*F* (1, 18) = 1.58, *P* = 0.225, *η*^2^ = 0.08) nor the group × time interaction was significant. *Post hoc* comparisons indicated a significant improvement from pre- to post-intervention (mean difference = 0.109, *P* < 0.001, 95% CI [0.077–0.141]). Both groups demonstrated comparable improvements over time (SVT: ES = 1.37; CON: ES = 0.88).

### Center of pressure COP test results

For COP displacement during the right-leg forward jump, significant main effects of time were observed in both the anterior–posterior (AP) and mediolateral (ML) directions (AP: *F* (1, 18) = 28.79, *P* < 0.001, *η*^2^ = 0.615; ML: *F* (1, 18) = 89.68, *P* < 0.001, *η*^2^ = 0.833).

No significant main effects of group were found (AP: *P* = 0.906; ML: *P* = 0.893), and no significant group × time interactions were observed (both *P >* 0.05).

*Post hoc* analyses revealed significant reductions in COP displacement in both groups. In the AP direction, both the SVT group (mean difference = −2.865, *P* = 0.001, 95% CI [−4.417, −1.313]) and the CON group (mean difference = −2.740, *P* = 0.002, 95% CI [−4.292, −1.188]) improved significantly. In the ML direction, both the SVT group (mean difference = −3.840, *P* <  0.001, 95% CI [−4.703, −2.977]) and the CON group (mean difference = −1.664, *P* < 0.001, 95% CI [−2.527, −0.801]) also showed significant improvements.

Both groups exhibited small-to-moderate effect sizes (SVT: ES = 0.29–0.41; CON: ES = 0.25–0.48). Overall, improvements were slightly greater in the ML direction than in the AP direction (Sarvari et al., 2024).

For COP displacement during the right-leg lateral jump to the right, significant main effects of time were observed in both AP and ML directions (AP: *F* (1, 18) = 90.13, *P* < 0.001, *η*^2^ = 0.834; ML: *F* (1, 18) = 25.78, *P* < 0.001, *η*^2^ = 0.589).

No significant main effects of group were found (AP: *P* = 0.873; ML: *P* = 0.751), and no significant group × time interactions were observed (all *P >* 0.05).

*Post hoc* analyses indicated significant improvements in both groups. In the AP direction, the SVT group (mean difference = −3.255, *P* < 0.001, 95% CI [−4.052, −2.458]) and the CON group (mean difference = −1.841, *P* < 0.001, 95% CI [−2.638, −1.044]) improved significantly. In the ML direction, both the SVT group (mean difference = −2.715, *P* <  0.001, 95% CI [−4.126, −1.304]) and the CON group (mean difference = −2.109, *P* = 0.006, 95% CI [−3.520, −0.698]) also showed significant reductions. Effect sizes were small-to-moderate (SVT: ES = 0.33–0.45; CON: ES = 0.00–0.28), with relatively greater improvements observed in the AP direction.

For COP displacement during the right-leg lateral jump to the left, significant main effects of time were observed in both AP and ML directions (AP: *F* (1, 18) = 145.92, *P* < 0.001, *η*^2^ = 0.890; ML: *F* (1, 18) = 141.86, *P* < 0.001, *η*^2^ = 0.887).

No significant main effects of group were found (AP: *F* (1, 18) = 0.069, *P* = 0.796, *η*^2^ = 0.004; ML: *F* (1, 18) = 0.060, *P* = 0.809, *η*^2^ = 0.003), and no significant group × time interactions were detected (AP: *P* = 0.789–0.803; ML: *P* = 0.794–0.839).

*Post hoc* comparisons revealed significant improvements in both groups. In the AP direction, the SVT group (mean difference = −3.040, *P* < 0.001, 95% CI [−4.185, −1.895]) showed greater reductions than the CON group (mean difference = −2.505, *P* <  0.001, 95% CI [−3.650, −1.360]). Similarly, in the ML direction, reductions were slightly larger in the SVT group (mean difference = −2.680, *P* < 0.001, 95% CI [−3.327, −2.033]) compared with the CON group (mean difference = −2.583, *P* < 0.001, 95% CI [−3.230, −1.936]). Effect sizes were small-to-moderate (SVT: ES = 0.40–0.42; CON: ES = 0.25–0.28). Although between-group differences were not statistically significant, the SVT group showed consistently greater improvements.

For COP displacement during the rotational jump, significant main effects of time were observed in both AP and ML directions (AP: *F* (1, 18) = 251.79, *P* < 0.001, *η*^2^ = 0.933; ML: *F* (1, 18) = 251.79, *P* < 0.001, *η*^2^ = 0.933).

No significant main effects of group were found (AP: *F* (1, 18) = 0.012, *P* = 0.915, *η*^2^ = 0.001; ML: *F* (1, 18) = 0.012, *P* = 0.915, *η*^2^ = 0.001), and no significant group × time interactions were observed (AP: *P* = 0.685–0.839; ML: *P* = 0.685–0.839).

*Post hoc* analyses demonstrated significant improvements in both groups. In the AP direction, the SVT group (mean difference = −4.880, *P* < 0.001, 95% CI [−5.516, −4.244]) showed greater reductions than the CON group (mean difference = −1.917, *P* < 0.001, 95% CI [−2.553, −1.281]). In the ML direction, the SVT group (mean difference = −6.820, *P* < 0.001, 95% CI [−7.929, −5.711]) also demonstrated larger improvements than the CON group (mean difference = −2.073, *P* < 0.001, 95% CI [−3.182, −0.964]).

Effect sizes were notably larger in the SVT group (AP: ES = 0.91 *vs.* 0.18; ML: ES = 0.58 *vs.* 0.16). Although interaction effects were not statistically significant, the consistently greater improvements in the SVT group suggest a potential advantage under rotational conditions.

## Discussion

### Effects of different balance training interventions on static balance in badminton athletes

The findings of this study showed that six weeks of SVT significantly improved static balance performance under visual deprivation conditions compared with CON. Although both groups demonstrated improvements, the SVT group exhibited greater gains, suggesting that SVT may be more effective for enhancing sport-specific balance ability. These results are consistent with previous findings (Lee et al., 2022), which reported improvements in both static and dynamic balance following SVT interventions. It should also be noted that the participants in this study were trained badminton athletes, and the relatively homogeneous performance level may have contributed to the sensitivity of detecting training effects. From a sensorimotor control perspective, postural stability is maintained through the integration of visual, proprioceptive, and vestibular inputs (Gribble, Hertel & Plisky, 2012; Paillard, 2017). Intermittent visual occlusion during SVT may challenge visual processing and promote greater reliance on non-visual sensory inputs. This process is often described as sensory reweighting, whereby the relative contribution of different sensory systems is adjusted (Haran & Keshner, 2008). However, as no direct neurophysiological measurements were conducted in the present study, these mechanisms should be interpreted as potential explanations rather than confirmed effects. In addition, SVT may provide a more challenging and variable training environment compared with conventional balance training, which could contribute to the observed improvements in static balance. Such training conditions may enhance the adaptability of postural control strategies under visually constrained situations.

### Effects of different balance training interventions on dynamic balance in badminton athletes

The present study showed that SVT resulted in greater improvements in dynamic balance compared with conventional training, which is consistent with previous findings on balance training interventions in badminton athletes (Malwanage, Senadheera & Dassanayake, 2022). In the Y-Balance Test, the SVT group demonstrated significantly larger increases in reach distances in the anterior, posteromedial, and posterolateral directions for both dominant and non-dominant legs. These findings suggest that SVT may be more effective in enhancing dynamic postural control during single-leg tasks. In the closed-eye stepping test, both groups improved performance following the intervention, although no significant between-group differences were observed. This suggests that both training approaches can enhance balance under visually deprived conditions, while SVT may provide additional benefits for more complex dynamic tasks. One possible explanation for these findings is that intermittent visual occlusion during SVT may increase reliance on proprioceptive and vestibular information (Proske & Gandevia, 2012), thereby influencing postural control strategies (Horak, 2006; Miao et al., 2024). However, these interpretations remain speculative, as no direct measurements of neuromuscular or sensory system function were included in this study. Previous studies have also reported that visual perturbation training can improve balance and movement performance in athletes (Mitrousis et al., 2023; Zwierko et al., 2024). These findings support the idea that training under altered visual conditions may enhance the adaptability of postural control in dynamic environments.

### Effects of different balance training interventions on landing stability in badminton athletes

Landing stability is essential for badminton athletes to ensure efficient transitions between movements. The results of the present study indicated that improvements in landing stability were significantly greater in the SVT group compared with the CON group. Specifically, the reduction in Dynamic Postural Stability Index (DPSI) suggests a faster return to a stable state following landing, reflecting improved dynamic stability. This finding is consistent with previous studies demonstrating that combined balance and plyometric training enhances landing control and change-of-direction performance in badminton athletes (Guo et al., 2021).

In addition, changes in center of pressure (COP) parameters indicate more precise control of body position during landing tasks. These findings suggest that SVT may contribute to improved postural regulation under dynamic conditions.

One possible explanation is that training under intermittent visual occlusion may challenge the sensory system and promote more adaptive movement strategies during landing. However, as no direct neuromuscular or neurophysiological measurements were conducted, these mechanisms remain speculative (Hoppeler, 2016; Snijders et al., 2015).

Previous studies have reported that stroboscopic training can improve visuomotor performance and reaction-related outcomes (Appelbaum et al., 2012), which may indirectly support improved landing control. Nevertheless, further research incorporating electromyographic or neurophysiological assessments is required to clarify the underlying mechanisms. These improvements may have practical implications for badminton performance, particularly in enhancing movement efficiency, reducing injury risk during landing, and facilitating faster transitions between strokes.

### Limitations

Several limitations of this study should be acknowledged. First, the sample size was relatively small; however, this is common in studies involving trained athletic populations due to strict inclusion criteria and limited availability. Second, all participants were male collegiate badminton athletes, which may limit the generalizability of the findings to other populations. Third, no direct neurophysiological or electromyographic measurements were conducted, preventing confirmation of the underlying mechanisms. Finally, sport-specific performance outcomes were not assessed, and future studies should investigate whether improvements in balance translate to on-court performance.

## Conclusions

In conclusion, six weeks of stroboscopic visual training significantly improved static balance, dynamic balance, and landing stability in collegiate badminton athletes compared with conventional balance training. These findings suggest that incorporating SVT into training programs may be an effective strategy for enhancing sport-specific postural control and movement stability.

From a practical perspective, these improvements may contribute to better movement efficiency, enhanced landing control, and faster transitions during high-intensity badminton play. Future research should further explore the underlying mechanisms and examine the transfer of these improvements to sport-specific performance.

##  Supplemental Information

10.7717/peerj.21464/supp-1 Supplemental Information 1 Trial protocol describing the study design and experimental procedures Detailed trial protocol outlining the study design, participant allocation, interventions, and outcome assessments.

10.7717/peerj.21464/supp-2 Supplemental Information 2 CONSORT 2010 checklist for the randomized controlled trial reported in this study

10.7717/peerj.21464/supp-3 Supplemental Information 3 Anonymized raw data supporting the analyses of stroboscopic balance training in collegiate badminton players

## Additional Information and Declarations

### Data Availability

The following information was supplied regarding data availability: The data is available in the Supplemental Files.

### Clinical Trial Registration

The following information was supplied regarding Clinical Trial registration: This study was not prospectively registered in a public trial registry because it involved a non-clinical, behavioral training intervention in healthy participants, and no medical, pharmaceutical, or patient-related outcomes were investigated. Ethical approval was obtained from the Research Ethics Committee of Beijing Sport University (Approval No. 2024486H).
