# Supplementary material for: Stroboscopic balance training enhances dynamic stability and postural control in collegiate badminton players: a randomized controlled trial
Source: PeerJ. 2026 Jun 26;14:e21464. doi: 10.7717/peerj.21464 (PMC13312999; doi:10.7717/peerj.21464)

Trial Protocol（Supplementary File S1）

1. Study Title

Stroboscopic Balance Training Enhances Dynamic Stability and Postural Control in Collegiate Badminton Players: A Randomized Controlled Trial

2. Trial Registration

This study was not registered, as it is a behavioral intervention and not a clinical trial.

3. Ethics Approval

Approved by the Research Ethics Committee of Beijing Sport University (Approval No. 2024486H).

All procedures complied with the Declaration of Helsinki.

4. Study Design

A single-blind, two-arm, parallel-group randomized controlled trial with 1:1 allocation ratio.

5. Participants

5.1 Inclusion Criteria

Male collegiate badminton athletes

Age 20–25

≥3 years badminton training

No lower-limb injury in previous 3 months

No conditions affecting balance

Not in major competition preparation

No caffeine/alcohol/medications affecting performance

Able to attend all sessions

5.2 Exclusion Criteria

Injuries during study

Non-compliance

Withdrawal from training or testing

5.3 Recruitment Period

May 2024, Beijing Sport University.

6. Randomization and Blinding

Randomization via random number table.

Participants assigned to SVT (n = 10) or CON (n = 10).

Blinding: Participants were blinded.

SVT group wore active stroboscopic eyewear.

CON group wore identical eyewear but switched off.

7. Interventions

7.1 SVT Group

Balance training + active stroboscopic eyewear

3 sessions/week, 30 min each, for 6 weeks

7.2 CON Group

Same training but eyewear inactive (transparent)

7.3 Training Components

Unstable-surface training

Dynamic balance tasks

Progressive overload principle

Standard technical–tactical training (5× /week)

8. Outcome Measures

8.1 Primary Outcome

Dynamic Postural Stability Index (DPSI) during jump-landing tasks

8.2 Secondary Outcomes

Center of Pressure (COP) displacement

Y-Balance Test composite scores

Closed-eye stepping test duration

Static single-leg balance performance

Measured at baseline and post-intervention (within 3 days).

9. Statistical Analysis

SPSS 25.0

Data as Mean ± SD

Two-way mixed-design ANOVA

Interaction effects analyzed with simple-effects tests

Effect sizes: partial η² and Cohen’s d

Significance level: P < 0.05

10. Adverse Events

No adverse events reported.

11. Protocol Availability

This Trial Protocol is submitted as Supplementary File S1.

CONSORT flow diagram ：


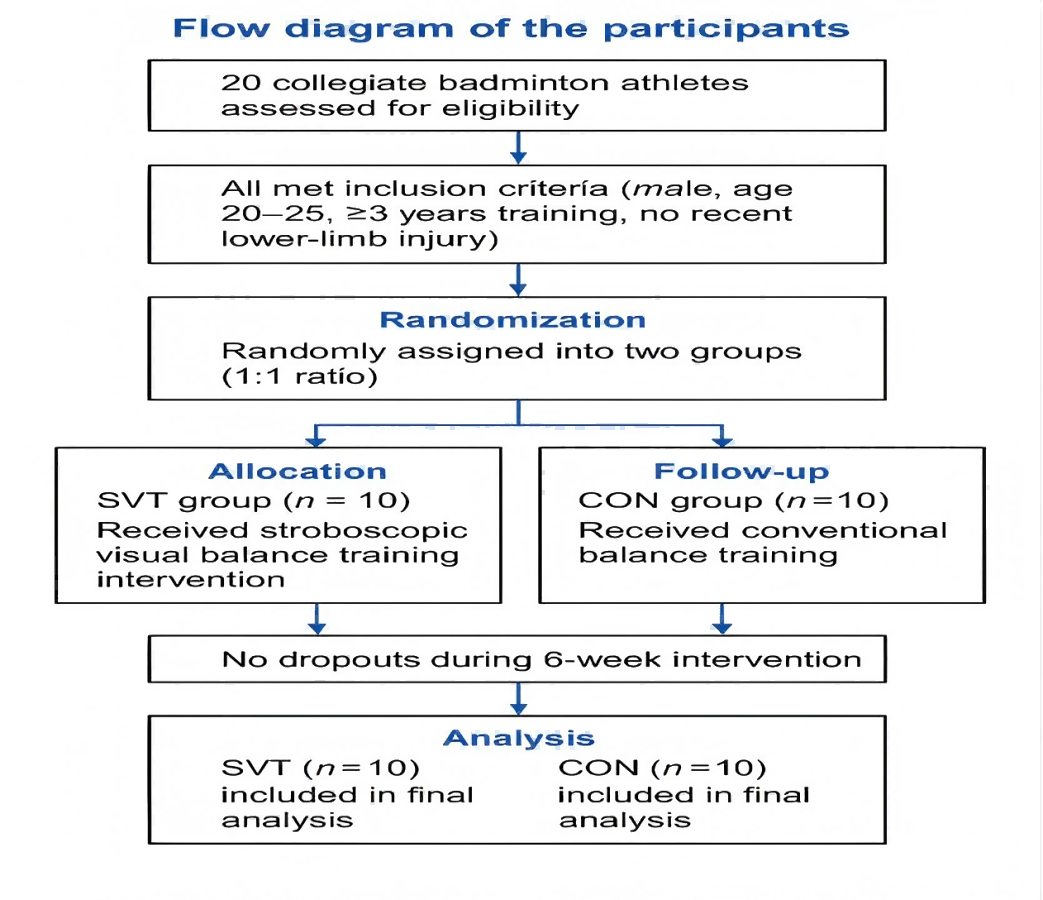

Supplement: Supplemental Information 1 — Detailed trial protocol outlining the study design, participant allocation, interventions, and outcome assessments. [file peerj-14-21464-s001.docx]
