# Supplementary material for: Stroboscopic balance training enhances dynamic stability and postural control in collegiate badminton players: a randomized controlled trial
Source: PeerJ. 2026 Jun 26;14:e21464. doi: 10.7717/peerj.21464 (PMC13312999; doi:10.7717/peerj.21464)
Supplement: Supplemental Information 2 [file peerj-14-21464-s002.docx]

# CONSORT 2010 Checklist (Completed)

Study: Stroboscopic Balance Training Enhances Dynamic Stability and Postural Control in Collegiate Badminton Players: A Randomized Controlled Trial

This checklist is completed based on the submitted manuscript.

| Section/Topic | Item No. | Checklist item | Reported on page/section |
| --- | --- | --- | --- |
| Title and Abstract | 1a | Identification as a randomized trial in the title | Title |
| Title and Abstract | 1b | Structured summary of trial design, methods, results | Abstract |
| Introduction | 2a | Scientific background and rationale | Introduction |
| Introduction | 2b | Specific objectives or hypotheses | Introduction |
| Methods | 3a | Description of trial design | Methods – Participants / Experimental Design |
| Methods | 3b | Important changes to methods after trial commencement | Not applicable |
| Methods | 4a | Eligibility criteria for participants | Methods – Participants |
| Methods | 4b | Settings and locations where data were collected | Methods – Testing Procedures |
| Methods | 5 | Interventions for each group | Methods – Experimental Design / Intervention Protocol |
| Methods | 6a | Pre-specified primary and secondary outcome measures | Methods – Testing Methods |
| Methods | 6b | Changes to trial outcomes after commencement | Not applicable |
| Methods | 7a | How sample size was determined | Methods – Participants |
| Methods | 7b | Interim analyses and stopping guidelines | Not applicable |
| Randomisation | 8a | Method used to generate random allocation sequence | Methods – Experimental Design |
| Randomisation | 8b | Type of randomisation; details of restriction | Methods – Experimental Design |
| Randomisation | 9 | Allocation concealment mechanism | Not applicable |
| Randomisation | 10 | Implementation | Methods – Experimental Design |
| Blinding | 11a | Blinding (who was blinded) | Methods – Experimental Design |
| Blinding | 11b | Similarity of interventions | Methods – Experimental Design |
| Statistical Methods | 12a | Statistical methods used to compare groups | Methods – Statistical Analysis |
| Statistical Methods | 12b | Methods for additional analyses | Not applicable |
| Results | 13a | Participant flow | Figure 1 (CONSORT flow diagram) |
| Results | 13b | Losses and exclusions after randomisation | Figure 1 |
| Results | 14a | Dates defining recruitment and follow-up | Methods – Participants |
| Results | 14b | Why the trial ended or was stopped | Not applicable |
| Results | 15 | Baseline demographic characteristics | Results / Table 1 |
| Results | 16 | Number of participants analysed | Results |
| Results | 17a | Outcomes and estimation | Results |
| Results | 17b | Binary outcomes | Not applicable |
| Results | 18 | Ancillary analyses | Not applicable |
| Results | 19 | Harms | Not applicable |
| Discussion | 20 | Trial limitations | Discussion |
| Discussion | 21 | Generalisability | Discussion |
| Discussion | 22 | Interpretation of results | Discussion |
| Other Information | 23 | Registration number and name of trial registry | Not applicable |
| Other Information | 24 | Where the full trial protocol can be accessed | Supplementary File S1 |
| Other Information | 25 | Sources of funding | Funding statement |
